# Time-course study of genetic changes in periodontal ligament regeneration after tooth replantation in a mouse model

**DOI:** 10.1038/s41598-024-66542-8

**Published:** 2024-07-05

**Authors:** Jun Ohshima, Shotaro Abe, Masayoshi Morita, Nobutake Tanaka, Masaya Yamaguchi, Mikako Hayashi

**Affiliations:** 1 Department of Restorative Dentistry and Endodontology, Osaka University Graduate School of Dentistry, 1-8 Yamadaoka, Suita, Osaka, 565-0871 Japan; 2 Bioinformatics Research Unit, Osaka University Graduate School of Dentistry, Osaka, Japan; 3 Bioinformatics Center, Research Institute for Microbial Diseases, Osaka University, Osaka, Japan; 4 Department of Microbiology, Osaka University Graduate School of Dentistry, Osaka, Japan; 5 Center for Infectious Diseases Education and Research, Osaka University, Osaka, Japan

**Keywords:** Gene expression, Periodontitis, Dental diseases, Gingivitis

## Abstract

This research focused on analyzing gene expression changes in the periodontal ligament (PDL) after tooth re-plantation to identify key genes and pathways involved in healing and regeneration. Utilizing a mouse model, mRNA was extracted from the PDL at various intervals post-replantation for RNA sequencing analysis, spanning from 3 to 56 days. The results revealed significant shifts in gene expression, particularly notable on day 28, supported by hierarchical clustering and principal component analysis. Gene ontology (GO) enrichment analysis highlighted an upregulation in olfactory receptor and G protein-coupled receptor signaling pathways at this time point. These findings were validated through reverse transcription-quantitative PCR (RT-qPCR), with immunochemical staining localizing olfactory receptor gene expression to the PDL and surrounding tissues. Moreover, a scratch assay indicated that olfactory receptor genes might facilitate wound healing in human PDL fibroblasts. These results underscore the importance of the 28-day post-transplant phase as a potential “tipping point” in PDL healing and regeneration. In conclusion, this research sheds light on the potential role of olfactory receptor genes in PDL regeneration, providing a foundation for developing new therapeutic approaches in tooth replantation and transplantation, with broader implications for regenerative medicine in oral health.

## Introduction

Complete dislocation of teeth is commonly observed in clinical practice, occurring in approximately 0.5 to 16% of dental trauma cases^1^. Avulsion is one of the most severe traumatic injuries, causing significant damage to periodontal tissues, especially in children and adolescents. In such cases, the most appropriate treatment is replantation^2^. However, root resorption poses a significant challenge. This resorption is primarily attributed to total or partial loss of the periodontal ligament, damage to the cementoblast layer, and the patient’s inflammatory response^3,4^. Consequently, the re-planted tooth might be lost, compromising the esthetic, functional, and psychological aspects of the patient’s life. Besides tooth dislocation from trauma, there are other scenarios requiring similar surgical techniques, such as intentional replantation for conditions such as refractory apical periodontitis^5,6^, root fractures^7,8^, or transplanting wisdom teeth to other locations^9,10^.

The periodontal ligament (PDL) is a connective tissue that anchors the tooth in the alveolus and is crucial for the success of these treatments^11^. While numerous clinical studies have detailed the process of alveolar bone synthesis and PDL healing post-tooth replantation^3,12^, few have examined the precise mechanisms of PDL regeneration^13–15^. PDL establishment requires a complex interplay of genes, proteins, and signaling pathways. However, comprehensive data of gene expression in the PDL over time is scarce and the genetic mechanisms influencing the healing and regeneration of dissociated PDL are largely unexplored, impeding the development of more effective therapeutic strategies.

This study therefore seeks to shed light on gene expression in the PDL during its healing and regeneration phases. We aimed to fill the existing knowledge gap to pave the way for novel drug discoveries and therapeutic strategies in tooth transplantation and replantation therapy.

## Results

### Comprehensive gene expression analysis of re-planted periodontal ligament

Optimal healing of a damaged periodontal ligament is observed when an extracted tooth is promptly replanted into its original extraction socket. Initially, we developed a mouse model for intentional tooth replantation with reference to previous studies^16^. We then extracted mRNA from the periodontal ligament at various intervals post-replantation and conducted a comparative transcriptome analysis using RNA-seq. This analysis was carried out on days 3, 7, 14, 28, and 56 post-replantation. Micro-computed tomography analysis was also conducted to verify post-replantation bone pathology (Supplementary Fig. S1). In the replanted teeth, the expansion of the periodontal lumen peaked at 28 days and was then decreased at 56 days.

**Figure 1:**
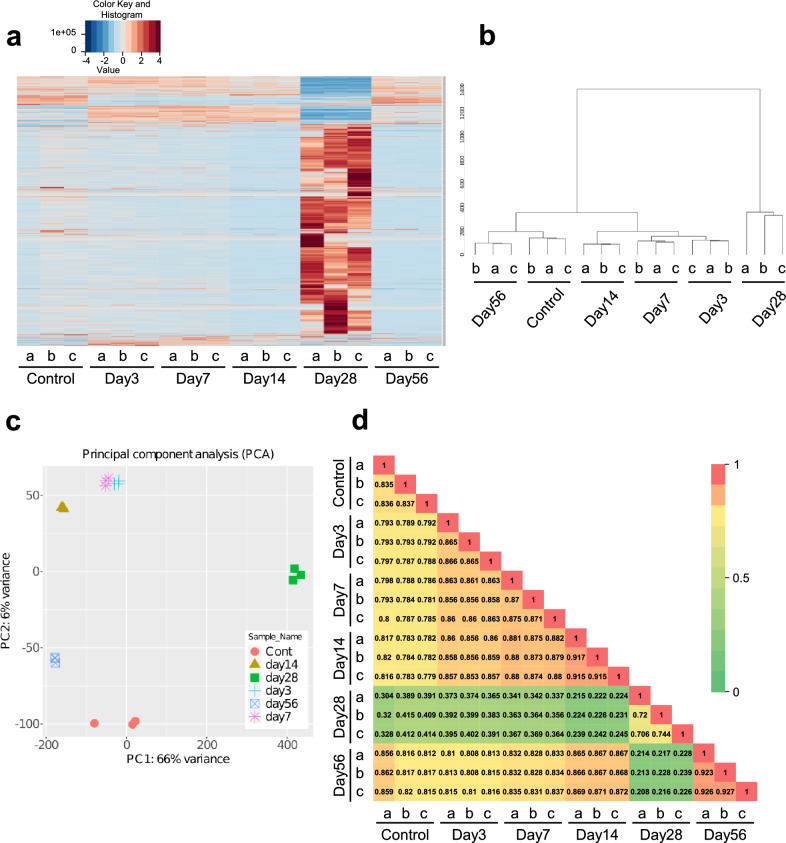
Comprehensive analysis of gene expression in periodontal ligament following tooth re-plantation. The maxillary first molar of an 8-week-old male mouse was extracted and re-planted immediately afterward. n = 3 per experimental group. (**a**) Heatmap illustrating the gene expression profiles in the periodontal ligament at various time points following re-plantation. Color scale: red, high relative expression; blue, low relative expression. (**b**) Hierarchical clustering of samples by Transcripts Per Million (TPM) values performed for all detected genes. (**c**) Principal component analysis plot showing the clustering of samples based on their gene expression profiles. Each dot represents an RNA-seq sample. (**d**) Correlation matrix comparing overall gene expression profiles. The closer the Pearson’s correlation coefficient is to 1, the greater the similarity between the samples. Bar: 0 (Green) to 1 (Red).

RNA-seq was performed to determine the impact of tooth extraction and replantation on gene expression within the periodontal ligament. The expression patterns were visualized using heatmaps of all identified genes. Pronounced shifts in gene expression patterns were evident in samples post-28 days (Fig. 1a). Hierarchical clustering analysis further corroborated that the 28-day samples were distinctly separate from the other groups (Fig. 1b). Principal component analysis showed that these samples were clustered within each group, with clear differences in genetic profiles among the groups (Fig. 1c). Additionally, the Pearson correlation coefficient (PCC) between each sample was computed and represented on a heatmap. The PCC values between the control group and each sample at 3, 7, 14, and 56 days were high (PCC > 0.77), while the sample at 28 days had a distinctly lower PCC value (PCC < 0.42) (Fig. 1d).

### Functional characterization of changes in gene expression at 28 days post-replantation

**Figure 2:**
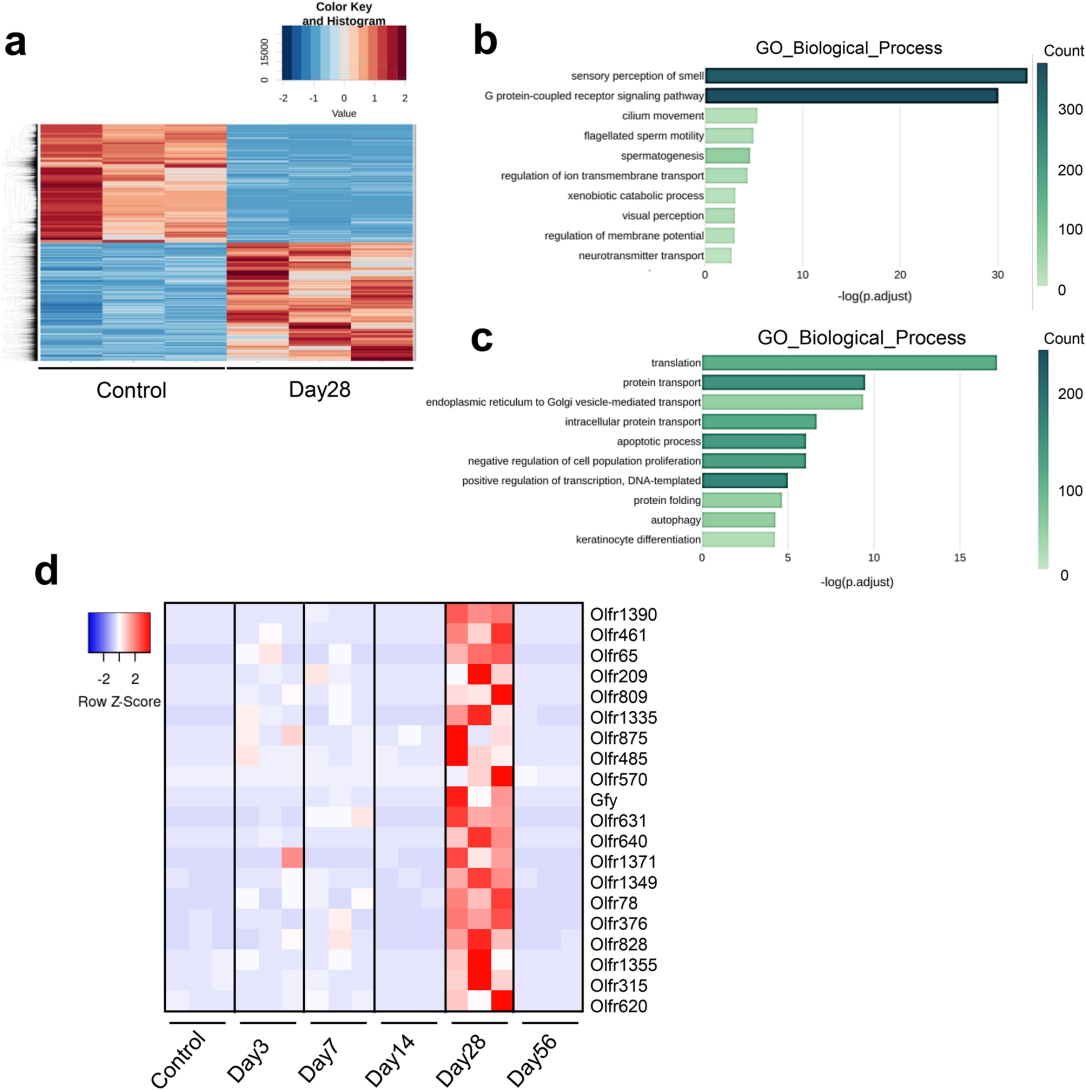
Functional alterations in gene expression at 28 days post-replantation. (**a**) Heatmap of differentially expressed genes and relative log expression-normalized values compared with controls. Each row represents one gene and each column represents one sample. The color scale shown at the top-left illustrates the relative expression level of genes across all samples: blue represents an expression level below the mean, and red represents an expression higher than the mean. (**b–c**) Gene Ontology (GO) enrichment using biological process terms analysis was performed to identify molecular functions of the transcriptome in the periodontal ligament on the 28th day after replanting. (**b**) The top 10 enriched GO biological process terms for transcripts up-regulated at 28 days post-replant [adjusted *P* < 0.05 and abs (log2FoldChange) > 0]. Adjusted *P* values sorted individual GO terms. (**c**) The top 10 enriched GO biological process terms for transcripts down-regulated at 28 days post-replant [adjusted *P* < 0.05 and abs (log2FoldChange) > 0]. Adjusted *P* values sorted individual GO terms. (**d**) Heatmap showing RNA-Seq differential expression data for the top 20 genes associated with sensory perception of smell (GO:0007608) across different time points. *AB*, Alveolar bone; *D*, dentin; *PDL*, periodontal ligament.

To discern the molecular functions of the altered transcriptome 28 days after replantation, we generated a heatmap by converting the expressed genes and relative log expression-normalized values, which were then compared with controls, into Z-scores. Subsequently, we conducted a GO enrichment analysis using biological process terms (Fig. 2a–c). Pathways that were significantly enriched were identified using genes with adjusted *P* < 0.05 and abs (log2FoldChange) > 0. The top 10 upregulated and downregulated GO category pathways are depicted in Fig. 2b, c, respectively.

Intriguingly, as illustrated in Fig. 2b, the predominant pathways that exhibited increased expression on day 28 were related to sensory perception of smell (GO:0007608) and the G protein-coupled receptor signaling pathway (GO:0007186). Inflammatory pathways, such as the inflammatory response (GO:0006954) and neutrophil chemotaxis (GO:0030593), saw an increase up to 14 days post-replantation (Supplementary Fig. S2a–d). Conversely, the pathways that witnessed a decline in expression on day 28 included translation (GO:0006412), protein transport (GO:0015031), and endoplasmic reticulum to Golgi vesicle-mediated transport (GO:0006888), indicating an anticipated reduction in cellular activity. By day 14, pathways such as keratinization (GO:0031424) and cell–cell adhesion (GO:0098609) had diminished (Supplementary Fig. S3a-c), indicating a weakened epithelial barrier function. Interestingly, the sensory perception of smell (GO:0007608) and G protein-coupled receptor signaling pathway (GO:0007186) were decreased in the periodontal ligament on days 14 and 56, and these signaling pathways were transiently upregulated at 1 month after transplantation (Supplementary Fig. S3c–d). We visualized the expression of the top 20 genes in the sensory perception of smell category (GO:0007608) that showed increased expression on day 28, across other time points using a heatmap. We observed a significant surge on day 28, followed by a return to basal levels at 56 days post-replantation (Fig. 2d).

### Quantitative PCR validation of representative genes for odor sensory pathways

**Figure 3:**
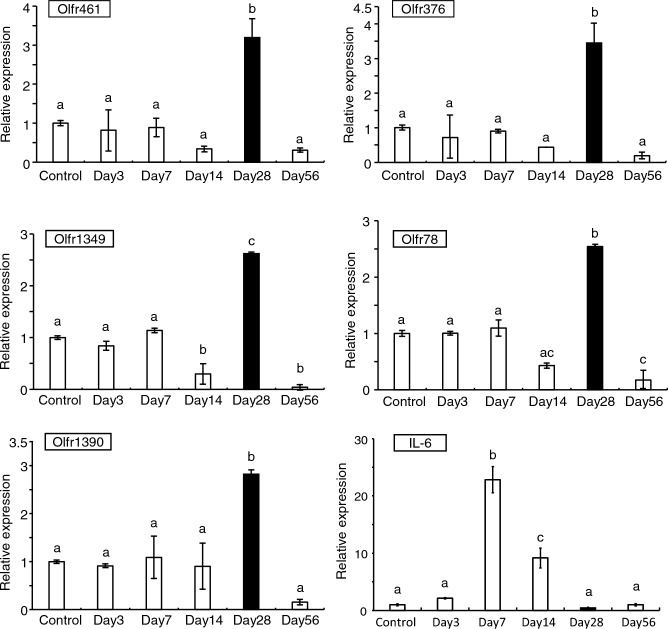
Quantitative PCR (RT-qPCR) validation of olfactory receptor genes and *Il-6* expression. Periodontal ligament from mice was sampled at different time points post-replantation and the isolated RNA was subjected to cDNA synthesis and RT-qPCR. Gene expression values were normalized relative to *Gapdh*. Values are the means ± standard deviation (SD) of triplicates and different lowercase letters (**a**–**c**) represent a significant difference among the six samples at *P* value < 0.05. Data are representative of three independent experiments.

To confirm the odor sensory pathways identified in the RNA-seq analysis, we conducted RT-qPCR to assess the expression of several olfactory receptor (OR)-related genes. RT-qPCR results of expression patterns for genes *Olfr461*, *Olfr376*, *Olfr1349*, *Olfr78*, and *Olfr1390* were aligned closely with those from the RNA-seq analysis (Fig. 3). This supports the transient expression of OR genes in the periodontal ligament as revealed by RNA-seq. Furthermore, qPCR analysis of IL6 expression, a cytokine inflammatory response marker, demonstrated that its expression peaked on day 7 and subsequently declined, indicating the resolution of inflammation.

### Immunohistochemical staining of OR genes

**Figure 4:**
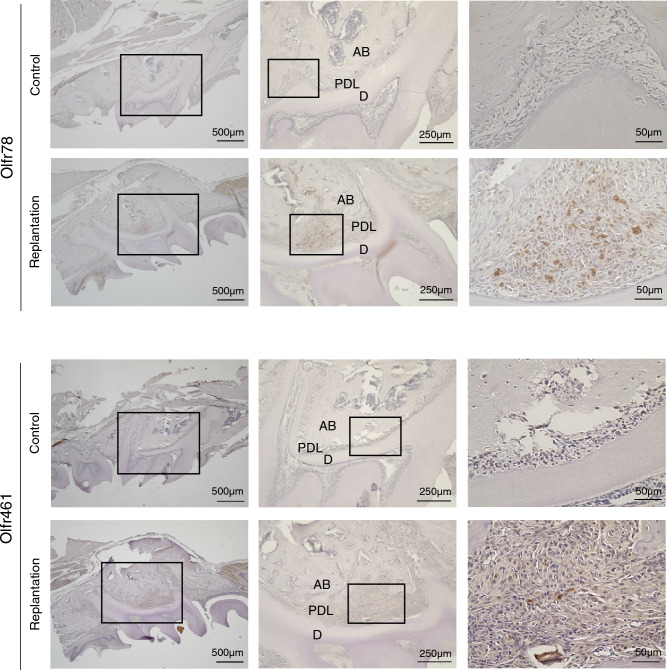
Immunohistochemical staining of control samples and collected samples 28 days post-replantation with each olfactory receptor-specific antibody. (**a**) Olfr78 and (**b**) Olfr461 are both strongly expressed in the periodontal ligament of replanted teeth. Rectangles in images denote enlarged sites. *AB*, Alveolar bone; *D*, dentin; *PDL*, periodontal ligament.

To pinpoint the location of OR gene expression, immunohistochemical staining was conducted on samples taken 28 days post-replantation. Notably, for both Olfr78 and Olfr461, there was a marked increase in expression within the PDL of the replanted teeth and the adjacent tissue in the experimental group. In contrast, the control group (which did not undergo replantation) exhibited minimal reactivity (Fig. 4).

### Wound healing and analysis of olfactory gene expression over time

**Figure 5:**
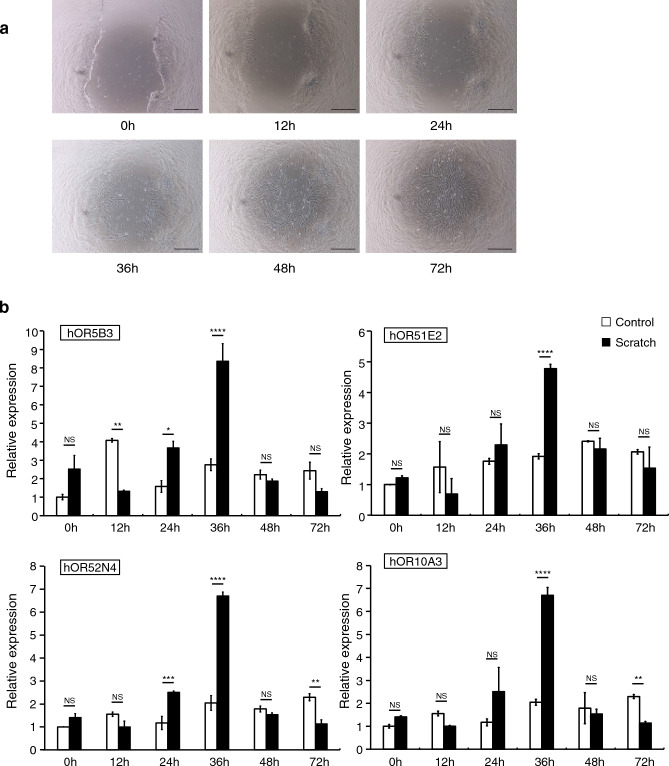
Time-course analysis of wound healing and olfactory receptor gene expression. (**a**) Images from a scratch assay at different time points. Human periodontal ligament fibroblasts were plated and wounded with a P1000 pipette tip. The width of the scratch decreased over time and was almost closed after 48–72 h. (Scale = 100 µm) (**b**) Olfactory receptor gene expression profiles over time during wound healing. NS, not significant; *P < 0.05, **P < 0.01, ***P < 0.001, ****P < 0.0001. Data are representative of three independent experiments.

To investigate the role of the OR gene in the wound-healing process, we conducted a scratch assay using human periodontal ligament fibroblasts. The width of the scratch diminished over time and was nearly fully closed between 48 and 72 h (Fig. 5a). Furthermore, we analyzed the expression of OR genes to understand their patterns during wound healing. As the healing advanced, the expression of representative genes, such as *OR5B3*, *OR51E2*, *OR52N4*, and *OR10A3* increased, with a notable surge peaking at 36 h (Fig. 5b).

## Discussion

We conducted an in-depth analysis of gene expression changes in periodontal tissues, focusing on the periodontal ligament of teeth that were replanted shortly after extraction. At 28 days post-implantation, there was a notable increase in gene expression related to sensory perception (primarily olfaction) and G protein-coupled receptor signaling pathways. This indicates a potential role of ORs in the healing and regeneration of the periodontal ligament. Moreover, validation through wound healing assays indicated a transient activation of these genes during scar healing.

Drawing from histological examinations in prior animal studies, we designed our analysis to span five time points: 3 days (bleeding, thrombus, and exudate), 1 week (collagen binding and vascular formation), 2 weeks (new bone, cementum, and periodontal ligament formation), 4 weeks (realignment of periodontal ligament fibers, vascular repair, and nerve regeneration), and 8 weeks (healing)^17^. In previous studies, genetic analysis of genes involved in healing and regeneration of the periodontal ligament after replantation was only performed at the short time point of 7 days^18^. This study used the latest next-generation sequencing technology to comprehensively analyze gene expression in the PDL after replantation to give a deeper understanding of the intricate processes underpinning PDL healing and regeneration.

RNA-seq facilitates a comprehensive and quantitative assessment of the entire transcriptome^19^. The biological significance of the differentially expressed genes derived from RNA-seq is discerned through GO analysis^20^. This provides a deeper insight into the dynamic response of periodontal tissue post-tooth implantation. Comparison of the differential gene expression profiles of the periodontal ligament at various time points shows the transcriptome profile at 28 days after replantation to be significantly changed (Fig. 1). Intriguingly, when enrichment analysis was conducted to estimate pathways with notably increased expression on day 28, genes involved in OR and G protein-coupled receptor (GPCR) signaling pathways were predominantly present in the replanted teeth.

ORs are traditionally recognized to be localized on the plasma membrane of olfactory neurons in the olfactory epithelium. These receptors detect specific odor molecules and convert them into electrical signals. However, ORs are also expressed in a variety of tissues outside of the olfactory epithelium^21,22^. Ectopically expressed ORs have diverse functions, including sperm chemotaxis^23^, regulating metabolism in the kidney and pancreas^24,25^, and modulating inflammatory responses in macrophages^26^. Despite these findings, many endogenous ligands for ORs remain unidentified, making it challenging to speculate on the role of these receptors in non-olfactory tissues, such as the periodontal ligament^27^. No studies have specifically addressed the function of ORs in oral tissues. Interestingly, a recent transcriptome analysis revealed that OR expression increased in human periodontal ligament stem cells (hPDLSCs) treated with resveratrol, a natural phytoalexin that promotes tissue repair^28^. Resveratrol significantly enhances osteogenic potential by upregulating over 20 ORs, indicating a potential role for ORs in hPDLSC-driven bone regeneration. The elevated expression of ORs in the periodontal ligament during replantation might indicate activation of periodontal ligament stem cells. Studies aimed at identifying external ligands for ORs in specific cell types may shed light on the underlying mechanisms.

While the direct interaction of OR-related genes in the healing process is not fully elucidated, OR, being classified under GPCR, suggests that these receptors play a crucial role. Our GO analysis also supports the involvement of GPCR in periodontal ligament regeneration post-replantation. G protein-coupled receptors (GPCRs) are a diverse family of receptors that mediate a wide range of cellular responses. GPCRs are classified into different subtypes (Gs, Go, Gq, etc.), each with distinct downstream signaling pathways and functional outcomes. For instance, Gs-coupled receptors, of which ORs are part of this family, typically activate adenylate cyclase, increasing cyclic AMP (cAMP) levels. cAMP regulates various cellular functions and influences cell proliferation, differentiation, and apoptosis^29^. In contrast, Gq-coupled receptors activate phospholipase C, leading to the production of inositol trisphosphate (IP3) and diacylglycerol (DAG). This results in increased intracellular calcium levels and activation of protein kinase C, which also affects cell proliferation and differentiation^30^. Go-coupled receptors are generally involved in regulating ion channels and inhibiting other signaling pathways. These different GPCR pathways can lead to varied phenotypic effects on periodontal ligament cells, including proliferation, differentiation, and apoptosis, playing crucial roles in cell fate determination^31^. The specific roles of these GPCR subtypes in periodontal ligament regeneration remain to be fully elucidated. Future research should focus on dissecting these pathways to identify potential therapeutic targets for enhancing tissue regeneration. Understanding how different GPCR subtypes interact and regulate the healing and regeneration of the periodontal ligament may lead to the development of novel therapeutic approaches to maximize the efficacy of replantation and transplantation treatments.

We delineated differential gene expression profiles post-tooth replantation. Inflammatory pathways, such as inflammatory response (GO:0006954), and neutrophil chemotaxis (GO:0030593), increased up to 14 days after replantation (Supplementary Fig. S2a–d). These results are consistent with cytokine responses during the early healing phases^18,32^. The RT-qPCR data for interleukin 6 (*Il-6*), a pivotal cytokine that orchestrates the inflammatory response, revealed a peak in expression on day 7, which subsequently tapered off. This indicates that inflammation in the periodontal tissue is predominant in the initial stages and wanes as healing progresses (Fig. 3). Although the host inflammatory response is important as an initial defense mechanism against tissue damage and infection in the periodontal tissue of re-planted teeth^4^, persistent inflammation may lead to tissue destruction.

Genes associated with keratinization and epithelial barrier functionality declined in expression in the periodontal ligament up to 14 days post-replantation (Supplementary Fig. S3a–d). Oral tissue injuries heal more swiftly than skin injuries, characterized by rapid re-epithelialization and remodeling that curtails scar formation^33,34^. However, the swift migration of epithelial cells to the injury site might stymie the proliferation and differentiation of progenitor cells essential for cementum, PDL, and bone maturation, jeopardizing the equilibrium of the periodontal ligament^35^. Therefore, the observed suppression of keratinization and epithelial barrier function in the re-planted periodontal ligament may play an important role in preventing epithelial tissue infiltration into the periodontal ligament to foster new tissue development. In particular, after the inflammatory response subsides, an environment is created in which new cells can easily proliferate. The diminished barrier function during this phase might enhance the efficient delivery of vital cell components and nutrients, thereby bolstering tissue restoration and regeneration.

The initial increase in gene expression observed at 3 and 7 days post-replantation is considered to be part of the early inflammatory response and healing mechanisms. During this stage, the immune system is activated as an early phase of wound healing, releasing numerous cytokines and growth factors. These molecules are upregulated to control tissue damage and initiate the repair process. This initial response subsides with the downregulation observed at 14 days, marking the resolution of the acute inflammatory phase and the transition to the proliferative phase of tissue repair.

The significant upregulation observed at 28 days post-replantation represents a critical 'tipping point' in the healing and regeneration process. During this period, tissue remodeling is actively occurring, with the generation of new cells and the formation of the extracellular matrix progressing. Notably, the increased expression of genes related to OR and GPCR signaling pathways suggests enhanced cell-to-cell communication and efficient tissue regeneration. This indicates that 28 days post-replantation is a crucial time point for the most effective regeneration of the periodontal ligament.

This study employed a mouse model; therefore, the direct applicability of the findings to humans remains uncertain. Additionally, the specific cell types in periodontal tissue that exhibit heightened OR expression are yet to be identified. Nevertheless, our data reveal that some ORs are expressed in primary human periodontal ligament fibroblasts, raising the possibility that their transient elevated expression may play an important role in wound healing. The universality of these results should be approached with prudence given the study’s limited scope. However, these insights can pave the way for innovative therapeutic approaches in tooth replantation and transplantation.

We did not consider the prognosis of the re-planted tooth, because no interventions, such as removal or drying of the periodontal ligament, were undertaken post-extraction. Moreover, while root canal treatment is typically recommended within two weeks following replantation^2^, this procedure was not executed in our study because the subjects were mice. Consequently, the genetic status observed on the 28th day might be influenced by inflamed pulp, potentially communicated through dentin tubules.

The results of our study showed that the expression of genes related to the OR and GPCR signaling pathways was significantly increased 28 days after replantation, indicating that these pathways may play an important role in PDL healing and regeneration. Developing new drugs targeting these receptor pathways is expected to lead to treatments that promote the PDL healing process and improve replantation success rates. For example, screening compounds that enhance OR and GPCR activity could be used to identify drugs that promote PDL cell proliferation and differentiation. In addition, local administration of drugs that enhance OR and GPCR activity may be considered to promote PDL regeneration and shorten healing time. Clinically, considering that 28 days after replantation is a "turning point" in PDL healing and regeneration, monitoring biomarkers related to the OR and GPCR signaling pathways may lead to identifying the optimal treatment timing and method for individual patients.

In conclusion, we have revealed intricate patterns of gene expression throughout the healing and regeneration processes of the periodontal ligament, with notable transient activation of OR genes. These insights may contribute to the quality of tooth replantation and transplantation treatment and to the further understanding of regenerative medicine.

## Methods

### Intentional replantation

All animal experiments were conducted with the approval of the Animal Experimentation Committee of the Graduate School of Dentistry, Osaka University (R-02-003-0), and complied with the ARRIVE 2.0 guidelines. All methods were performed following the relevant guidelines and regulations. Animals were housed under specific pathogen-free conditions and maintained on a 12-h light/dark cycle. Eight-week-old male C57BL/6 J mice were injected intraperitoneally with sodium pentobarbital (SomnoPentyl®; Kyoritsu Seiyaku) at a dose of 30 mg/kg as a general anesthetic. Carprofen (Rimadyl®; Pfizer) was administered at a dose of 3 mg/kg to alleviate postoperative pain. The tooth surfaces and surrounding tissues were cleaned with an alcohol-soaked cotton ball. The right upper first molar was then extracted using auricular forceps (52-113-10; Daiichi Medical). After extraction, we ensured there was no damage to the periodontal tissue or root fractures before immediately re-planting the tooth in its original location. The re-planted maxillary right first molar and the maxillary left first molar (used as a control group) were evaluated after 3, 7, 14, 28, and 56 days.

### RNA sequencing (RNA-seq)

Experimental animals were euthanized using an intraperitoneal overdose of sodium pentobarbital (200 mg/kg, IP). The right and left maxillary first molars were then extracted. The periodontal ligament tissue from the extracted teeth was detached using a micro excavator, and the tissue fibers were disrupted with a homogenizer. RNA was then extracted using the FastGene RNA Basic Kit (NIPPON Genetics). Total RNA samples were sent to Rhelixa Corporation for Bioanalyzer quality control (Agilent) and next-generation sequencing analysis. Library preparation was conducted using total RNA from each sample and the SMART-Seq HT kit (Clontech). Sequencing was performed on the Illumina NovaSeq 6000 system, yielding approximately 26 million 150 bp paired-end (PE) sequence reads per sample. These PE FASTQ files underwent trimming using Trimmomatic. Sequence reads were mapped with HISAT2, and reads were counted per gene using featureCounts. Fragments per kilobase of transcript per million mapped reads (FPKM), FPKM-upper quartile (FPKM-UQ), and transcripts per million (TPM) values were then calculated. These data were imported into various bioinformatics analysis programs, such as iDEP and stats, to visualize the data^36^.

### Quantitative real-time PCR (RT-qPCR)

cDNA was synthesized from extracted total RNA using a High-Capacity cDNA Reverse Transcription Kit (Thermo Fisher Scientific). Real-time PCR was conducted on a 7300 Fast Real-Time PCR System (Thermo Fisher Scientific) using the Power SYBR® Green Master Mix (Thermo Fisher Scientific). Values were normalized to the expression level of glyceraldehyde-3-phosphate dehydrogenase (*GAPDH*) in each sample. Primer sequences can be found in Supplementary Table S1.

### Immunohistochemistry

After euthanasia via an intraperitoneal overdose of sodium pentobarbital, the animals underwent concentrated flow fixation in a 4% paraformaldehyde phosphate buffer (Nacalai Tesque). The maxillary bone containing the test teeth was extracted, and the soft tissues were removed. Following immersion and fixation in a 4% paraformaldehyde phosphate buffer for 24 h at 4 °C, the teeth were demineralized with Calkitox (Fujifilm Wako) over 3 days. Post-demineralization, specimens were dehydrated through an ascending alcohol series, embedded in paraffin, and serial sections of 5 μm thickness were prepared using a rotary microtome (RM 2155; Leica). Immunochemical staining followed the VECTASTAIN Elite ABC Kit (Vector Laboratories) protocol. Before primary antibody application, sections were incubated in 10% goat serum to block nonspecific antigens. The primary antibodies used were against Olfr78 (1:32, ab140907; Abcam) and Olfr461 (1:500, OSF00031W; Osenses). After an overnight reaction with the primary antibodies at 4 °C, sections were rinsed and exposed to biotinylated secondary antibodies. Antibody staining was visualized using VECTASTAIN Elite ABC reagent and diaminobenzidine solution (ImPACT® DAB; Vector Laboratories). Nuclei were stained using Meyer’s hematoxylin solution. Samples underwent dehydration and clarification with a 70% to 100% ethanol series and xylene, were mounted using MOUNT-QUICK (Daido Sangyo), and observed under a fluorescence microscope (BZ-X800; KEYENCE).

### Micro-computed tomography

Changes in the re-planted teeth and periodontal bone volume were evaluated using micro-computed tomography (R-mCT2; Rigaku). Experimental animals were anesthetized through an intraperitoneal injection of sodium pentobarbital (30 mg/kg), and images were captured with a tube voltage of 90 kV, a tube current of 160 μA, and a slice width of 5 μm. The horizontal cross-section of the maxillary first molar was defined as the XY plane, the sagittal cross-section as the YZ plane, and the frontal cross-section as the ZX plane.

### Scratch wound healing assay

Primary human periodontal ligament fibroblasts were sourced from ScienCell Research Laboratories (#2630). Cells were cultured in Dulbecco’s modified Eagle’s medium (Nacalai Tesque) supplemented with 10% fetal bovine serum (Nichirei Bioscience), 100 U/ml penicillin, and 0.1 mg/ml streptomycin (Nacalai Tesque). The culture conditions were maintained at 37 °C, 5% CO_2_, and 100% humidity. Cells were seeded at 3 × 10^5^ cells/well in 6-well plates and incubated for 24 h. Subsequently, two vertical scratches were made using a P1000 pipette tip. The medium was refreshed to eliminate any unattached cells, and cell samples were harvested at intervals of 0, 12, 24, 36, 48, and 72 h. At each interval, images of the wound region were captured, and total RNA was isolated.

### Statistical analysis

Student’s t-test was used to determine differences between two groups. One-way ANOVA was used to test for differences between three or more groups, and Tukey’s test was used for multiple comparison tests. All analyses were conducted using Prism software (GraphPad), and *P* < 0.05 was deemed significant.

### Supplementary Information

Supplementary Information.

## Data Availability

The data have been deposited with links to BioProject accession number PRJDB 16,893 in the DDBJ BioProject database.
