# Supplementary material for: Time-course study of genetic changes in periodontal ligament regeneration after tooth replantation in a mouse model
Source: Sci Rep. 2024 Jul 5;14:15502. doi: 10.1038/s41598-024-66542-8 (PMC11226448; doi:10.1038/s41598-024-66542-8)
Supplement: Supplementary file 1 — Supplementary Information. [file 41598_2024_66542_MOESM1_ESM.pdf]

Supplementary Fig. S1

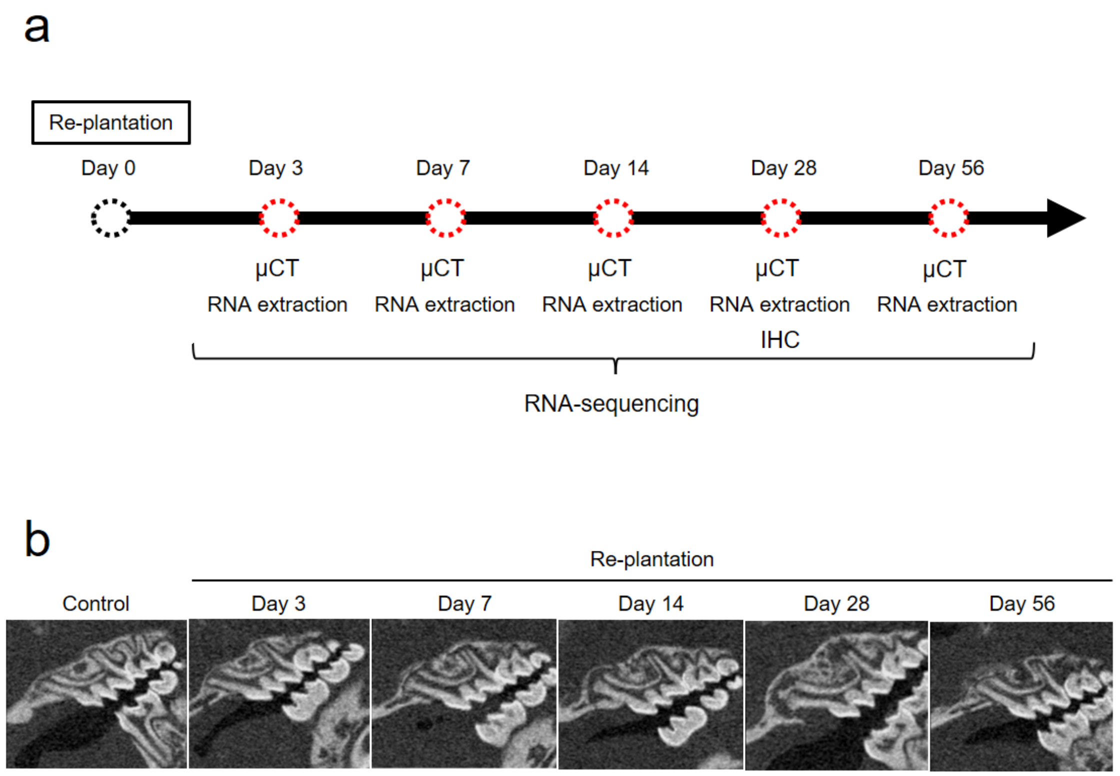

Supplementary Fig. S1. Experimental flow and micro-CT analysis of bone pathology. (**a**) The timeline of the experiment after replantation is shown. (**b**) Representative micro-computed tomography (µCT) images of the molars. To evaluate bone pathology, this analysis was performed at 3 days and 1, 2, 4, and 8 weeks after replantation. Data are representative of three independent experiments

Supplementary Fig. S2

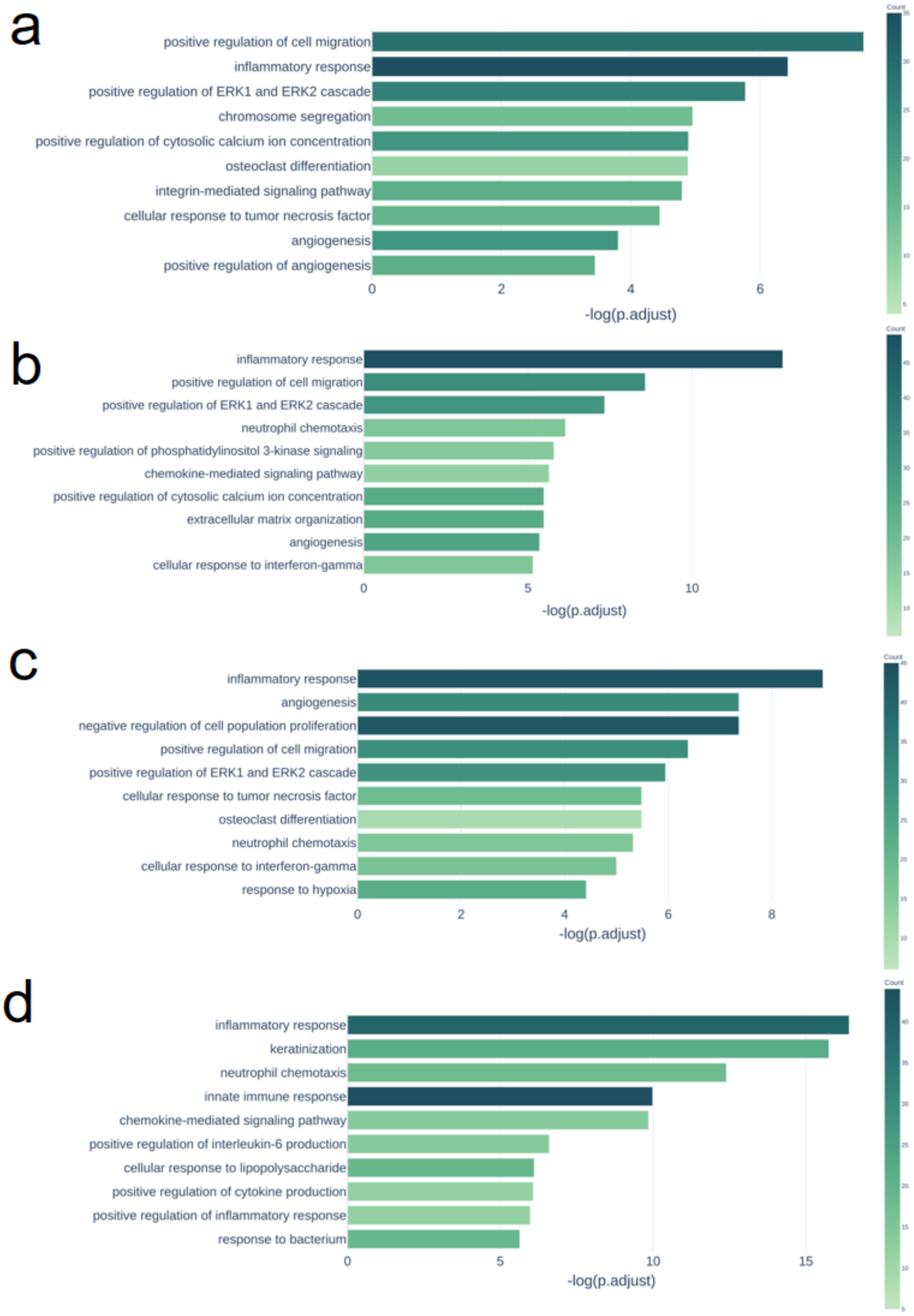

Supplementary Fig. S2. Gene Ontology (GO) term enrichment using biological process terms analysis for upregulated transcripts in periodontal ligament after replantation. The top 10 GO biological process terms for transcripts with up-regulated expression at 3 days (**a**), 1 week (**b**), 2 weeks (**c**), and 8 weeks (**d**) post-replantation. [adjusted $p < 0.05$ and abs (log2FoldChange) > 0]. Adjusted p-values sorted individual GO terms.

Supplementary Fig. S3

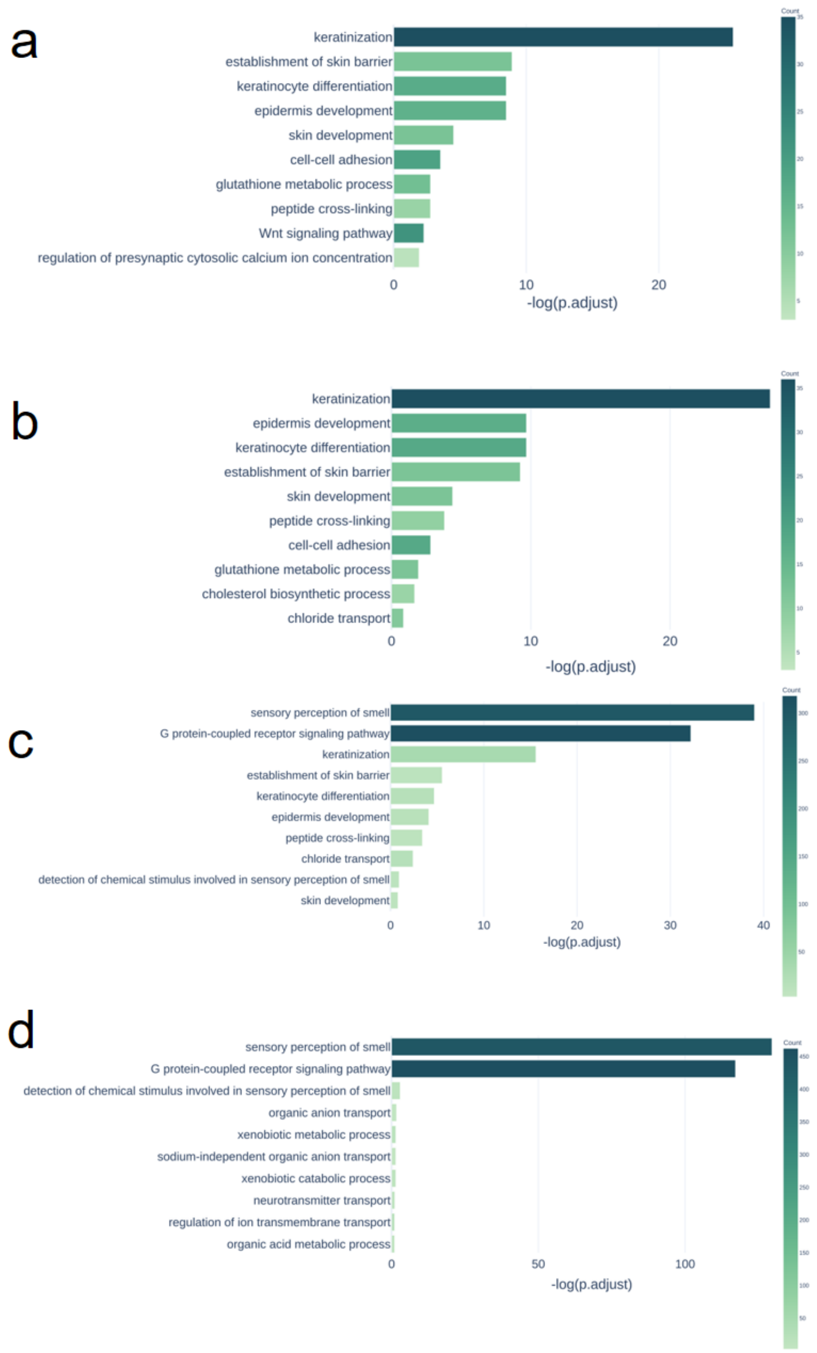

Supplementary Fig. S3. Gene Ontology (GO) term enrichment using biological process terms analysis for downregulated transcripts in periodontal ligament after replantation. The top 10 GO biological process terms for transcripts with down-regulated expression at 3 days (**a**), 1 week (**b**), 2 weeks (**c**), and 8 weeks (**d**) post-replantation. [adjusted $p < 0.05$ and abs (log2FoldChange) > 0]. Adjusted p-values sorted individual GO terms.

Supplementary Table S1. Primer sequences for real-time quantitative polymerase chain reaction.

Species	Primer name	Sequence (5' → 3')
Mouse	Gapdh_F	CATCACTGCCACCCAGAAGACTG
	Gapdh_R	ATGCCAGTGAGCTTCCCGTTCAG
	Olfr461_F	GTATGTGGCTGTCTGCAACCCT
	Olfr461_R	GCTGAAATGTGGCATAGACTGGC
	Olfr376_F	GTCATGGGAAGCCTGATCCTTG
	Olfr376_R	ACAGGTGGAGAAGGCTTTACGG
	Olfr1349_F	CACTCGTCTTCACCATCTGGCT
	Olfr1349_R	GCCTCGAAAACTCTGGTGTCTG
	Olfr78_F	GCAACTGCCTTCCAAGTCTGAG
	Olfr78_R	GGCTGTTTCCAAAACGGTGCAC
	Olfr1390_F	GCTGAAGACCAAGTCAATGGCAG
	Olfr1390_R	CCTTGCTCTCAGAATAGGTGCC
	Il-6_F	TACCACTTCACAAGTCGGAGGC
	Il-6_R	CTGCAAGTGCATCATCGTTGTTC
Human	GAPDH_F	GCTCAGACACCATGGGGAAG
	GAPDH_R	GAACATGTAAACCATGTAGTTGAGG
	OR5B3_F	ACCAGAAGCCTTTGTCCACCTG
	OR5B3_R	CAGGTGCCATTTTGTCTGTGTCC
	OR51E2_F	GCAACTGCCTTCCAAGTCAGAG
	OR51E2_R	GGCTGTTTCCAAAGCGGTGTAC
	OR52N4_F	CACCTTCACAGGGATGGAGTCT
	OR52N4_R	TCAGGAAGGTGGCAGTCCCAAC
	OR10A3_F	GATGCCATCAACTACTGGGAGAC
	OR10A3_R	CTTGGTTTCGGGTGAGTAGCCA
